# Determination of the absolute configuration of compounds bearing chiral quaternary carbon centers using the crystalline sponge method†
†Electronic supplementary information (ESI) available: Details of sample preparation and crystallographic analysis. CCDC 1492160–1492163 and 1537112. For ESI and crystallographic data in CIF or other electronic format see DOI: 10.1039/c7sc01524k



**DOI:** 10.1039/c7sc01524k

**Published:** 2017-05-16

**Authors:** Shiho Sairenji, Takashi Kikuchi, Mohamed Ahmed Abozeid, Shinobu Takizawa, Hiroaki Sasai, Yuichiro Ando, Kohsuke Ohmatsu, Takashi Ooi, Makoto Fujita

**Affiliations:** a Department of Applied Chemistry , School of Engineering , The University of Tokyo , 7-3-1 Hongo, Bunkyo-ku , Tokyo 113-8656 , Japan . Email: mfujita@appchem.t.u-tokyo.ac.jp; b JST ACCEL , 4-1-8 Honcho , Kawaguchi , Saitama 332-0012 , Japan; c The Institute of Scientific and Industrial Research (ISIR) , Osaka University , Mihogaoka , Ibaraki-shi , Osaka 567-0047 , Japan; d Institute of Transformative Bio-Molecules (WPI-ITbM) , Nagoya University , Nagoya 464-8602 , Japan

## Abstract

Determination of the absolute configuration of chiral tetra-substituted carbon centers is one of the most taxing steps in the enantioselective construction of this structural motif in asymmetric synthesis. Here, we demonstrate that the crystalline sponge method provides an effective way to crystallographically determine the absolute configuration of organic compounds bearing chiral quaternary carbons (including tetra-substituted ones) that are synthesized by recently developed enantioselective catalytic reactions.

## Abstract 2

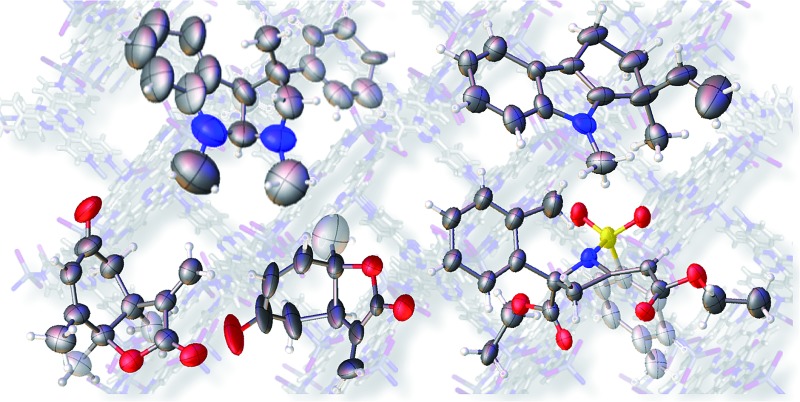
The absolute configuration of organic compounds bearing chiral quaternary carbons is determined by the crystalline sponge method.

## Introduction

Chiral quaternary carbon centers are important structural elements that are frequently found in biologically active natural compounds. The construction of chiral quaternary carbon centers by asymmetric synthesis is therefore a subject of intense research in current synthetic chemistry.^1^ Although many excellent methods have recently been developed for the chiral construction of quaternary carbons,^2^ the difficulty in determining the absolute configuration of these quaternary carbons in the products of interest remains a hurdle that is yet to be overcome. There are no empirical methods for predicting the absolute configuration of chiral quaternary carbons, and their configuration must therefore be determined by non-empirical methods, such as the Bijvoet method in single crystal X-ray structure analysis^3^ or vibrational CD analysis,^4^ unless the compounds can be stereospecifically derivatized from or to chiral compounds of known configuration. The non-empirical Bijvoet method is recognized as the most reliable method for absolute structure determination. However, the application of this method is limited because the target compound must be obtained as a high-quality single crystal and, in addition, the method requires incorporation of a heavy atom into the compound so that clear anomalous scattering can be observed.

The recently developed crystalline sponge method^5,6^ can completely overcome the limitations of the Bijvoet method. In the crystalline sponge method, sample crystallization is not required (the sample does not even need to be crystalline), and the incorporation of heavy atoms into the compound of interest is unnecessary because clear anomalous scattering is observed from the heavy atoms, Zn and I, in the host structure. The method has already demonstrated its usefulness in the determination of the absolute configuration of compounds with non-central chirality, for example axial and planar chirality,^7^ and of scarce natural products.^8,9^ Here, the crystalline sponge method is applied to the determination of the absolute configuration of compounds with quaternary and tetra-substituted chiral carbon centers that were obtained by efficient enantioselective reactions developed by the Sasai and Ooi groups (Table 1).^10–13^ The reactions, which are mostly promoted by chiral organocatalysts, give high ee values and are of current interest, but the absolute configurations of some of the products remain undetermined or have been only tentatively assigned using empirical methods. We show that the crystalline sponge method can easily, reliably and efficiently confirm the absolute configuration of these chiral products, and thus that the method is of great help in asymmetric synthesis. Throughout the study, we employed [(ZnI_2_)_3_(tpt)_2_(cyclohexane)_*x*_] (**1**, tpt = 2,4,6-tris(4-pyridyl)-1,3,5-triazine)^14^ as the crystalline sponge. Except for compound **2**, the absolute configurations of the chiral products in Table 1 have been undetermined or only empirically speculated in the previous studies. In the analysis of compound **6**, it is revealed that crystalline sponge discriminates methyl and vinyl groups at the stereogenic center.

**Table 1:** Enantioselective synthesis of compounds **2**, **4**, **6** and **8**, which possess quaternary or tetra-substituted chiral carbon centers

Run	Enantioselective reactions	Chiral catalyst or ligand
1^*a*^	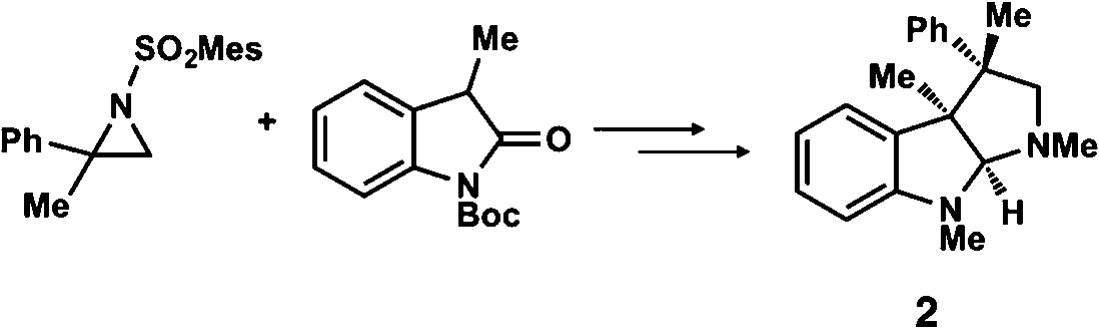	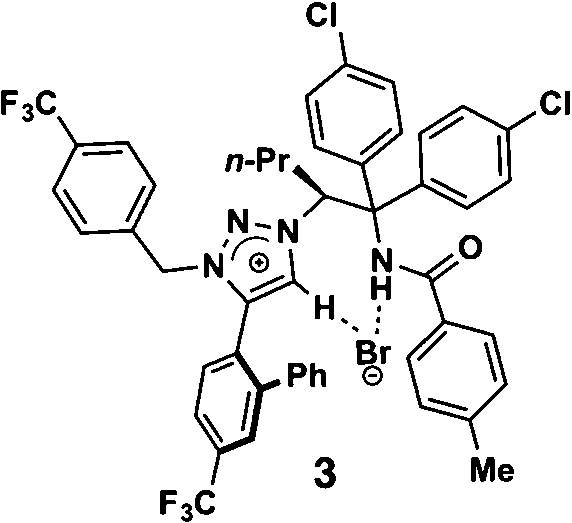
2^*b*^	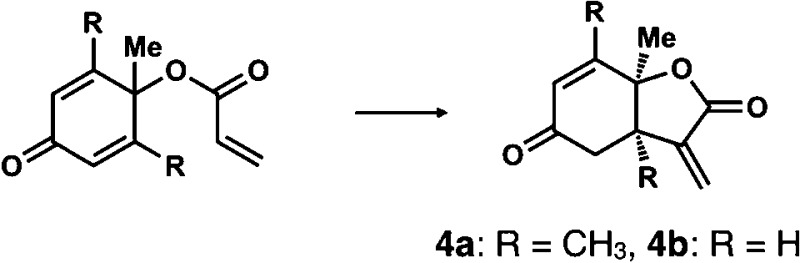	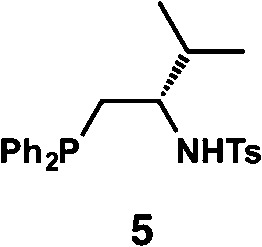
3^*c*^	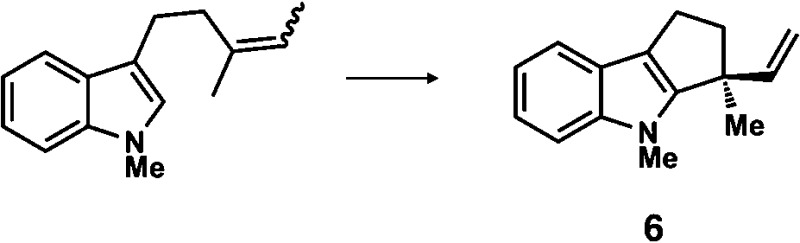	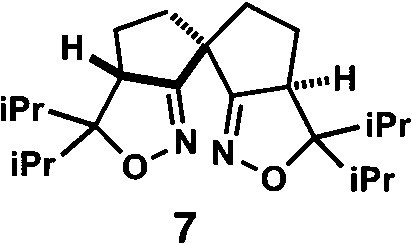
4^*d*^	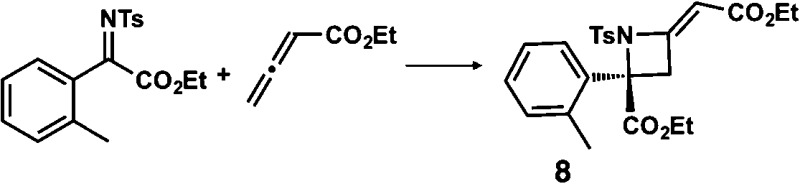	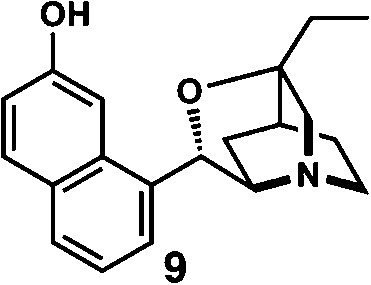

^*a*^Enantioselective synthesis of **2** reported by the Ooi group. (Ref. 10) Two adjacent chiral quaternary carbons are constructed by the diastereo- and enantioselective ring-opening alkylation of 2,2-disubstituted aziridines (scheme of run 1) with chiral organocatalyst **3**. The absolute configuration of **2** has been unambiguously determined by the crystallographic analysis (Bijvoet method) of its synthetic precursor.

^*b*^Enantioselective synthesis of **4** reported by the Sasai group (ref. 11) through the intramolecular Rauhut–Currier reaction (scheme of run 2) with chiral organocatalyst **5**. The absolute configuration of **4** has only been speculated by the comparison of the signs of specific rotation with that of a related compound of known configuration.

^*c*^Enantioselective synthesis of **6** developed by the Sasai group, (ref. 12) using the Pd(ii)-catalyzed Fujiwara–Moritani reaction (scheme of run 3) with chiral spiro bis(isoxazoline) ligand **7**. The absolute configuration of **6** has been undetermined.

^*d*^Enantioselective synthesis of **8** reported by the Sasai group (ref. 13) through the formal [2 + 2] cycloaddition of ketimines (scheme of run 4) with chiral organocatalyst **9**. The absolute configuration of **8** has only been speculated by the comparison of the signs of specific rotation with that of a related compound of known configuration.

## Results and discussion

The Ooi group has developed a highly enantioselective synthesis of pyrrolidinoindoline **2** (Table 1, run 1) that proceeds through the catalytic ring-opening alkylation of racemic 2,2-disubstituted aziridine and its reaction with 3-substituted oxindole.^10^ Such chiral quaternary carbon-bearing pyrrolidinoindoline derivatives are often found in natural products.^15^ The absolute configuration of the main enantiomer was established on the basis of the crystallographic analysis of its synthetic precursor, which contains a heavy atom and therefore allowed the Bijvoet method to be applied.^10^ The relative configuration of pyrrolidinoindoline **2** was also confirmed by crystallographic analysis of **2** itself. To confirm the utility of the crystalline sponge method in the absolute configuration determination of compounds containing chiral quaternary carbon atoms, we directly determined the absolute configuration of **2** using this method. A sample of **2** with >99% ee was obtained by the asymmetric reaction shown in Table 1 (run 1) using chiral triazolium catalyst (*S*)-**3**, and then subjected to analysis by the crystalline sponge method.

**Fig. 1:**
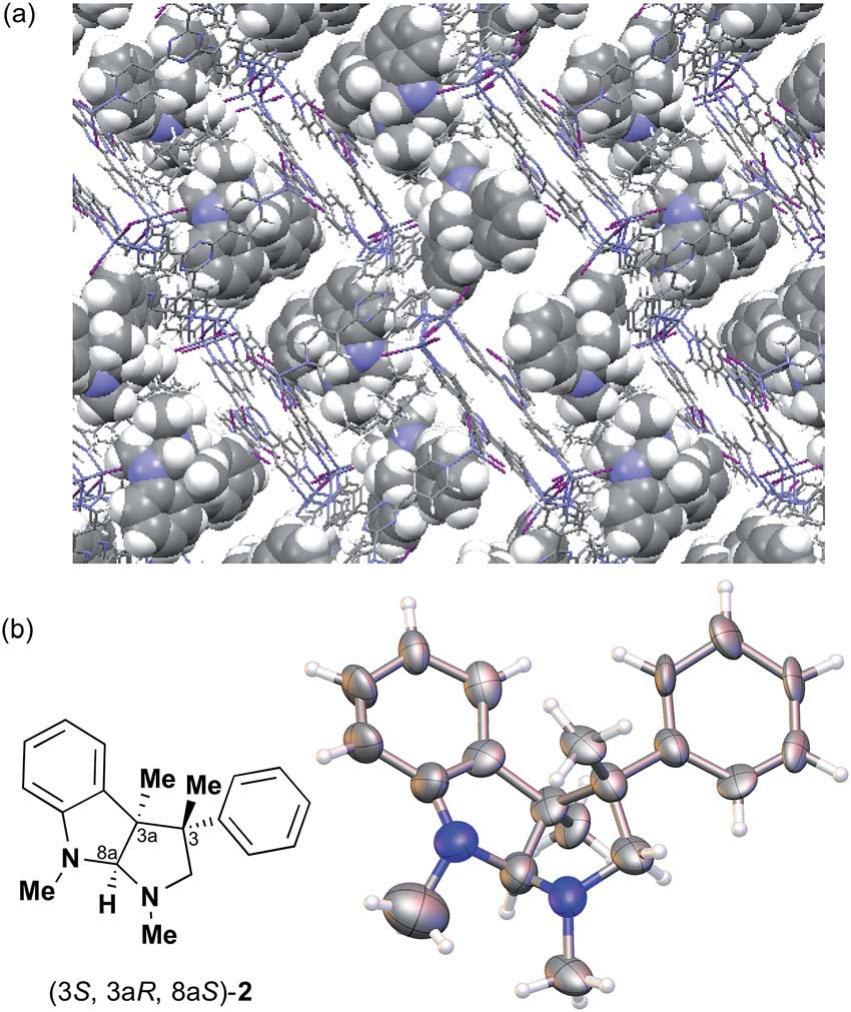
(a) Crystal structure of **2** incorporated in crystalline sponge **1** viewed along the *b* axis (**2**: spacefill model, others: stick model, disordered guests/solvents have been omitted for clarity). (b) ORTEP drawing of **2** at the 30% probability level.

A high quality single crystal (0.25 × 0.16 × 0.11 mm^3^) of crystalline sponge **1** was treated with a small amount of a colorless solution of **2** (5 μg) in cyclohexane/1,2-dichloroethane (9 : 1 v/v, 50 μL). After incubation at 50 °C for 1 d, the colorless crystal of **1** became red, which indicated that guest inclusion had occurred; the red color is characteristic of the host–guest interactions between **1** and **2**. The resultant crystal of **1·2** was subjected to X-ray diffraction on an in-house X-ray diffractometer with Cu Kα radiation. Owing to the efficient host–guest interactions, chirality was induced in the originally achiral host framework. The space group of the crystal changed from centrosymmetric *C*2/*c* to non-centrosymmetric *C*2 after inclusion of **2**; this was confirmed by the appearance of *h*0*l* (*l* = odd) reflections (extinction rule). After structural refinement, three molecules of **2** were found in the asymmetric unit cell. A value of 0.030(4) was obtained for the Flack parameter (calculated with the Parsons' quotients^3^), and we could therefore directly observe the absolute configuration of **2** as 3*S*,3a*R*,8a*S*, including two chiral quaternary carbon centers (Fig. 1). This confirms the previous determination of the absolute configuration of this structure using the Bijvoet method on the heavy atom-containing precursor. It is important to note that neither chemical derivatization nor crystallization of **2** itself were necessary to determine the absolute configuration of **2** with the crystalline sponge method, and the X-ray Bijvoet method could be directly applied even in the absence of heavy atoms in the compound.

We next analyzed compounds for which the absolute configuration had been only tentatively speculated by analogy with known compounds. Chiral compounds that include an α-alkylidene-γ-butyrolactone core are of great importance in medicinal chemistry,^16^ and the Sasai group has achieved the stereo- and enantioselective synthesis of one such compound, bicyclic γ-butyrolactone **4** (Table 1, run 2). This molecule is synthesized using the Rauhut–Currier reaction catalyzed by chiral organocatalyst (*S*)-**5**, which bears both Brønsted acid (–NHTs) and Lewis base (–PPh_2_) moieties.^11^ The stereochemical assignment of both the relative and absolute stereochemistry in **4** is not straightforward because this molecule contains two adjacent chiral carbon centers that do not bear hydrogen atoms. The *cis* stereochemistry of the lactone has been elucidated with a NOESY experiment, but its absolute configuration has so far only been speculated by comparing the optical rotation with that of a natural analog. We thus examined the absolute configuration of compound **4** using the crystalline sponge method.

**Fig. 2:**
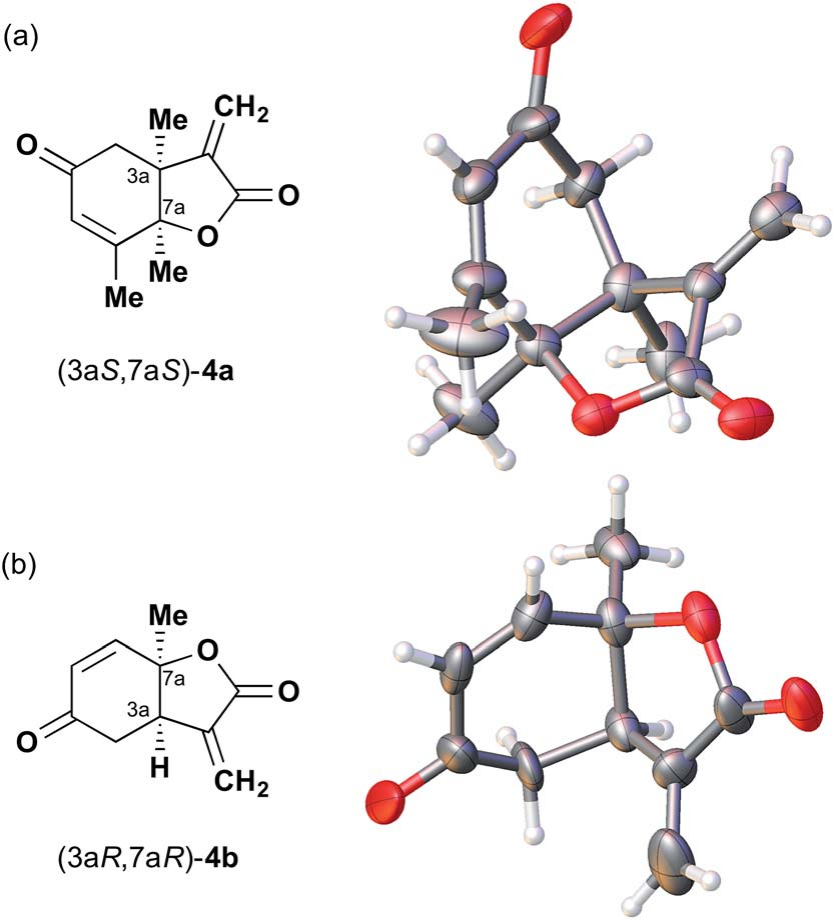
Crystal structure of (a) (3a*S*,7a*S*)-**4a** and (b) (3a*R*,7a*R*)-**4b** determined by the crystalline sponge method (ORTEP diagrams drawn at the 30% probability level).

After separating both enantiomers of **4a** from the racemic mixture by chiral HPLC with a Daicel Chiralpak IC column, we applied the crystalline sponge method to the first fraction. This fraction contained the minor enantiomer, shown in Table 1 (run 2). Guest soaking of **4a** was performed at 50 °C for 2 d. The structure of inclusion crystal **1·4a** was solved in the *C*2 space group with a Flack parameter (Parsons) of 0.008(6) (Fig. 2a). The α-alkylidene-γ-butyrolactone core of **4a** was clearly observed, and the absolute configuration was determined to be 3a*S*,7a*S*.

Compound **4b** was analyzed in a similar fashion, and the first fraction obtained from the chiral HPLC separation was soaked into the crystalline sponge. In this case, the first fraction corresponded to the major enantiomer formed in the reaction. The absolute configuration of **4b** in the first fraction was determined to be 3a*R*,7a*R* [Flack parameter (Parsons): 0.06(2)] (Fig. 2b). From the analysis of the minor enantiomer of **4a** and the major enantiomer of **4b**, we concluded that the major enantiomers of the asymmetric Rauhut–Currier reaction with catalyst (*S*)-**5** have the 3a*R*,7a*R* configuration. This configuration did not match the speculation in the previous report,^11^ warning that the signs of specific rotations cannot provide a convincing reference for elucidating the absolute configurations of chiral compounds.

**Fig. 3:**
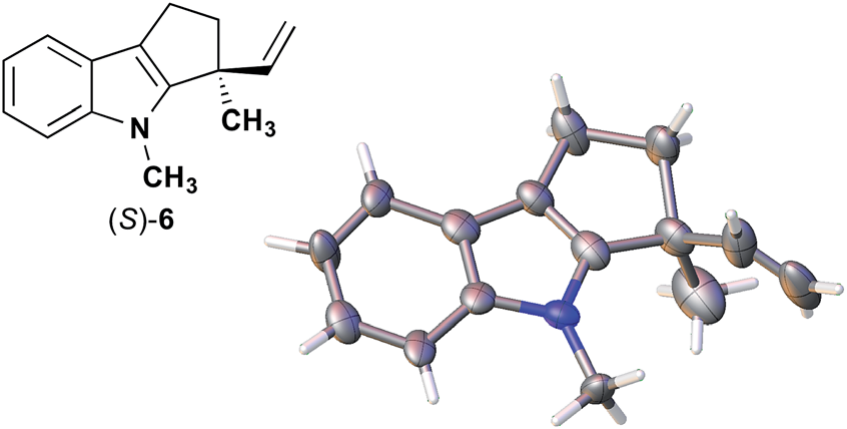
Crystal structure of (*S*)-**6** determined by the crystalline sponge method (ORTEP diagram drawn at the 30% probability level).

The next compound that we examined was chiral indole **6**, which was synthesized by the enantioselective Fujiwara–Moritani reaction^12^ (Table 1, run 3) using Pd(OCOCF_3_)_2_ (10 mol%) as the catalyst and (*P*,*R*,*R*)-**7** (15 mol%) as the chiral ligand. Because indole scaffolds are often found in biologically active natural products,^17^ this reaction is highly beneficial in terms of medicinal chemistry. After chiral HPLC purification using a Daicel Chiralpak IB column, the crystalline sponge method was applied to the major enantiomer of **6** that was formed. Guest soaking at 50 °C for 1 d with 5 μg of **6** worked well, and the guest-absorbed crystal was subjected to data collection. Space group conversion from *C*2/*c* to *C*2 was confirmed on the basis of the extinction rule. After structural refinement, seven independent molecules of **6** were clearly observed in the asymmetric unit cell. The stereogenic quaternary carbon center of all seven molecules was in the *S* configuration [Flack parameter (Parsons): 0.056(11)] (Fig. 3), which is consistent with the configurations of structurally related compounds.

In guest **6**, the chirality of the stereogenic quaternary carbon is defined by only C_1_ and C_2_ substituents (methyl and vinyl groups), which are often difficult to distinguish crystallographically. Before guest inclusion, the void of the crystalline sponge possesses an inversion center because of its *C*2/*c* symmetry. If the methyl and vinyl substituents were not distinguished, the loss of symmetry to the *C*2 space group would not take place, and overlapped electron density of the methyl and vinyl groups would be observed. Despite anticipating this result, we observed a clear change in the space group (*C*2/*c* to *C*2) and a sufficiently low Flack parameter, which indicated highly efficient discrimination between the methyl and vinyl substituents in the host–guest complexation. The electron density map (*F*_o_) showed the electron density of a terminal carbon atom (methyl group) and a C_2_ linkage with reasonable average bond lengths and angles for a vinyl group, and thus no overlap of the methyl and vinyl electron density. Such a high degree of molecular recognition in the void of the crystalline sponge is remarkable.

**Fig. 4:**
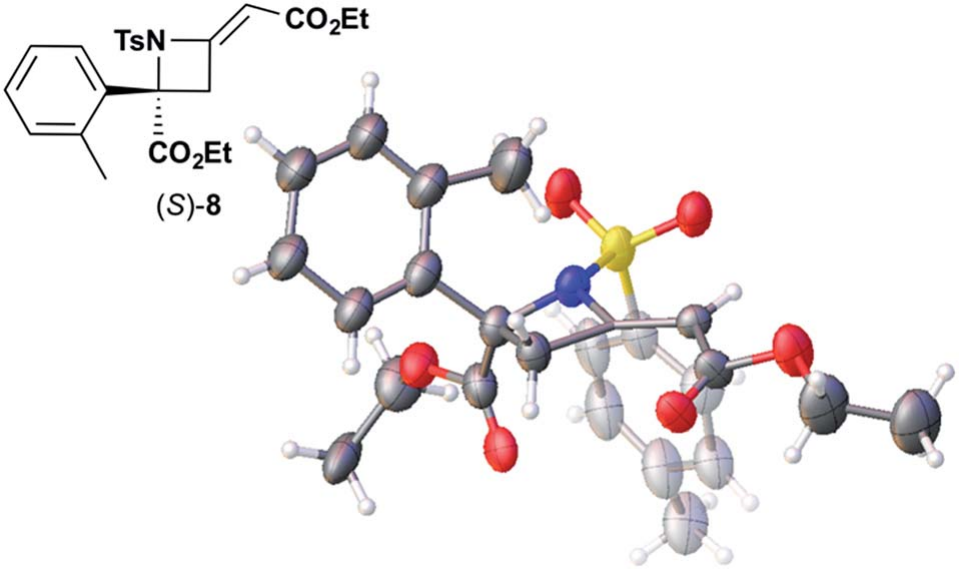
Crystal structure of (*S*)-**8** determined by the crystalline sponge method (ORTEP diagram drawn at the 30% probability level).

We also determined the absolute configuration of chiral azetidine **8**, which was synthesized by the enantioselective aza-Morita–Baylis–Hillman (aza-MBH) reaction between an a,β-unsaturated carbonyl compound and an imine (Table 1, run 4) with chiral organocatalyst **9**.^13^ The products of the aza-MBH reaction are highly functionalized allylic amines, which are useful building blocks for medicinal chemistry.^18^ A racemic sample of **8** was separated into its two enantiomers by chiral HPLC using a Daicel Chiralpak IC column, and the first peak, which was identified as the minor enantiomer, was subjected to the crystalline sponge analysis. The crystalline sponge method clearly showed that the absolute configuration of the minor enantiomer was *S* [Flack parameter (Parsons): 0.044(7)] (Fig. 4). Therefore, we confirmed that the enantioselective reaction shown in Table 1 (run 4) gives (*R*)-**8** as the major enantiomer. In this crystallographic analysis, the *E* stereochemistry of the trisubstituted olefin, which had previously only been speculated by NOE experiments, was also confirmed.

## Conclusions

The ability of the crystalline sponge method to establish absolute configurations of molecules has been demonstrated for chiral compounds bearing chiral quaternary or tetra-substituted carbon centers. Studies of the asymmetric synthesis of these compounds are, despite their frequent occurrence in biologically active compounds, often incomplete in that the absolute configuration of the major enantiomers are only speculated. Although one limitation of the crystalline sponge method is that not all molecules can be absorbed into the sponge, this is not a serious concern because the analysis of only a few samples among the many chiral compounds prepared in a synthetic study is usually enough to determine the enantioselectivity of the reaction. The crystalline sponge method is therefore certainly of great help in asymmetric synthesis studies for which, as for the compounds of interest in this study, no empirical rules are applicable for establishing the absolute configuration of the major enantiomers.
