# Supplementary material for: Determination of the absolute configuration of compounds bearing chiral quaternary carbon centers using the crystalline sponge method
Source: Chem Sci. 2017 May 16;8(7):5132–6. doi: 10.1039/c7sc01524k (PMC5615217; doi:10.1039/c7sc01524k)
Supplement: Supplementary file 1 [file SC-008-C7SC01524K-s001.pdf]

Electronic Supplementary Material (ESI) for Chemical Science.
This journal is © The Royal Society of Chemistry 2017

Supporting Information

Determination of the absolute configuration of compounds bearing chiral quaternary carbon centers using the crystalline sponge method

Shiho Sairenji,[a,b] Takashi Kikuchi,[a,b] Mohamed Ahmed Abozeid,[c] Shinobu Takizawa,[c] Hiroaki Sasai,[c] Yuichiro Ando,[d] Kohsuke Ohmatsu,[d] Takashi Ooi[d] and Makoto Fujita[a,b]

[a]*Department of Applied Chemistry, School of Engineering, The University of Tokyo, 7-3-1 Hongo, Bunkyo-ku, Tokyo, 113-8656, Japan*

[b]*JST ACCEL, 4-1-8 Honcho, Kawaguchi, Saitama, 332-0012, Japan*

[c]*The Institute of Scientific and Industrial Research (ISIR), Osaka University, Mihogaoka, Ibaraki-shi, Osaka, 567-0047, Japan*

[d]*Institute of Transformative Bio-Molecules (WPI-ITbM), Nagoya University, Nagoya, 464-8602, Japan*

Table of Contents

I. General Information

II. Sample Preparation and Structure Analysis

III. Reference

I. General Information

1. Reagents

Solvents and reagents were purchased from TCI Co., Ltd. and WAKO Pure Chemical Industries Ltd. and used without further purification.

2. Single crystal X-ray diffraction experiment

Single crystal X-ray diffraction data were collected on SuperNova (Rigaku Oxford Diffraction) diffractometer equipped with a micro-focus Cu $K\alpha$ radiation source (λ = 1.5418 Å), a high-sensitive CCD detector, and a low temperature system using cold nitrogen stream (100 K) (**1•2**, **1•4a**, **1•6**, and **1•8**), and on XtaLAB P200 (Rigaku Oxford Diffraction) diffractometer equipped with a fine-focus Mo $K\alpha$ radiation source (λ = 0.71073 Å), a hybrid pixel array detector (HPAD), and a low temperature system using cold nitrogen stream (93 K) (**1•4b**). Collected data were integrated, corrected, and scaled by the program CrysAlisPro. Empirical and numerical absorption corrections were applied in this process.

3. Crystal structure analysis

All crystal structures were solved using SHELXT ver. 2014/5[1] and refined using SHELXL ver. 2014/7[2] programs. All the non-hydrogen atoms were refined anisotropically. All the hydrogen atoms were grown using the proper HFIX command and refined isotropically using the riding model. Populations of the guests in the crystal were estimated from the least-square refinement of guest/solvent disorder model under the constraint that the sum of them should equal to 100%. Minimum number of restraints and constraints was applied for the least-square refinement, with reduced standard uncertainty in some cases for solvent molecules and host framework. Solvent cyclohexane, nitrobenzene, and 1,2-dichloroethane molecules in the pores were found in the difference electron density map and refined using the restraints and constraints (DFIX, DANG, SIMU, ISOR, DELU, FLAT, and AFIX 66). These molecules are expected to be severely disordered as a consequence of their high thermal motion and have an averaged structure of various geometry and orientation. This is a reason why some cyclohexane and 1,2-dichloroethane molecules are distorted to energetically-unfavorable structure, and the FREE command should be used for the refinement for

the solvents to avoid their close contact. Some “Alert A” notifications were found in the validation program CheckCIF. Those alerts are derived from short intermolecular contacts of hydrogen atoms between solvent molecule (cyclohexane and 1,2-dichloroethane) and the crystalline sponge framework, guest molecule, and other solvent molecule, and are unavoidable due to severe disorder of solvent molecules. The comments for the alerts are described in the CIFs using the validation response form (vrf).

II. Sample Preparation and Structure Analysis

1. Crystal 1•2

Sample preparation for 1•2

A solution of 5 μg of compound **2** in 5 μL of 1,2-dichloroethan was added to a microvial including a crystal of crystalline sponge **1** and 45 μL of cyclohexane. A screw cap of the microvial was pierced with a syringe needle and the solvent was slowly evaporated over 1 d at 50 °C. The resulting crystal was subjected to single crystal X-ray analysis.

Crystallographic data for 1•2

Crystal size: 250 × 164 × 107 μm^3, refined formula: $C_{122.98}H_{117.96}N_{28}Zn_6I_{12}$, formula weight ($M_r$): = 3903.19, brown block, crystal system: Monoclinic, space group $C2$, Z = 4, 33308 unique reflections merged from recorded 296791 ones (3.425° < θ < 76.286°) were used for structural analysis (R_{int} = 0.0804). Lattice parameters, R-factor on F^2 > 2σ(F^2), weighted R-factor, goodness-of-fit, and Flack parameter (calculated from 11435 Parsons' quotients) are follows: a = 34.8690(6) Å, b = 14.91350(10) Å, c = 31.5649(5) Å, β = 102.275(2)°, V = 16039.1(4) Å^3, R = 0.0689, wR = 0.2054, S = 1.026, χ = 0.030(4). Calculated density is 1.616 g·cm^{-3}. Linear absorption coefficient (μ) is 19.505 cm^{-1}. Residual electron density (max/min) is 1.342/−1.454 eÅ^{-3}. CCDC number 1492160.

Structure analysis for 1•2

The ORTEP diagram of the asymmetric unit of **1•2** is shown in Figure S1. Three guest molecules were found in the asymmetric unit. The occupancies of the guest molecules located at site ***A*** and ***B*** are fixed at 75%, which were determined as their thermal parameters have reasonable values. The other guest molecule is overlapped on the symmetrically generated one by the 2-fold rotation operation, and thus two guest molecules are disordered and occupy the inclusion site ***C*** with the total occupancy of 100%.

Some restraints should be applied for refinement of a disordered model. The guest at ***A*** was refined with applying DFIX (for the N–CH_3 bonds), and the geometries of the other two guests are related with that of guest ***A*** with applying SAME (for the whole

molecules). All the guest molecules were refined with applying SIMU (for the whole molecules). In addition, the guest at A was refined with applying DELU (for the Ph–C_3 bond).

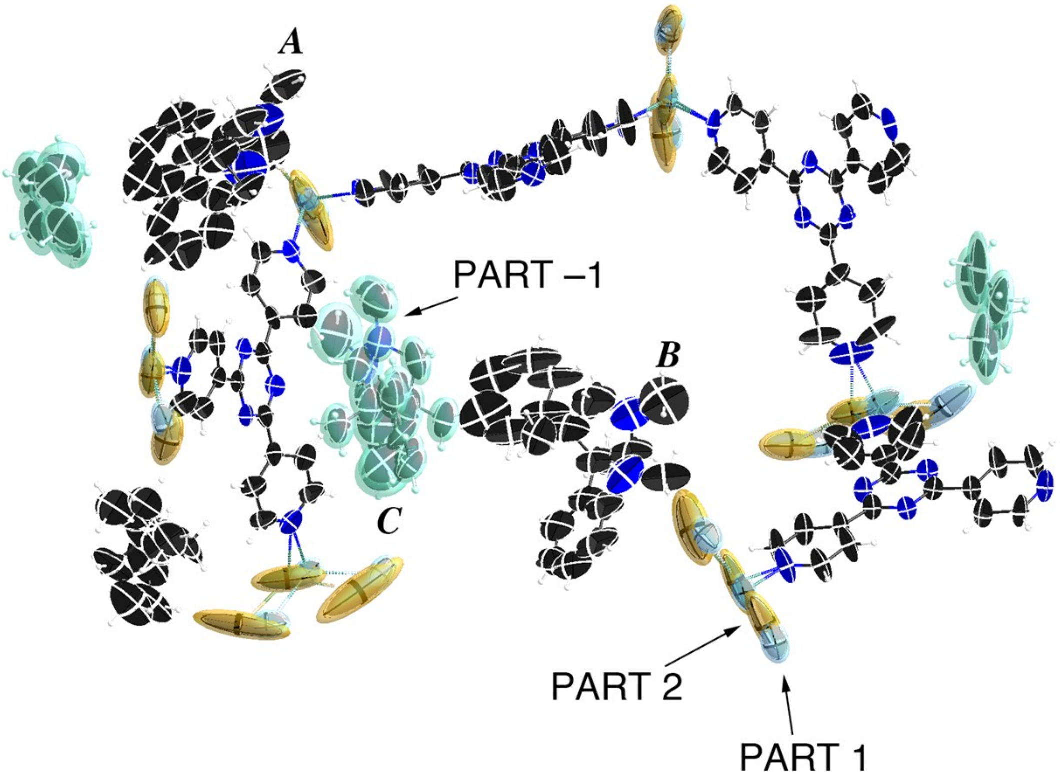

Figure S1. ORTEP diagram with 50% probability in the asymmetric unit of **1•2**. Enclosed atoms by the PART command in SHELXL were represented using the difference color code.

2. Crystal 1•4a

Sample preparation for 1•4a

A solution of 5 μg of compound **4a** in 5 μL of 1,2-dichloroethan was added to a microvial including a crystal of crystalline sponge **1**. The microvial was sealed with a screw cap and incubated at 50 °C for 2 days. The resulting crystal was subjected to single crystal X-ray analysis.

Crystallographic data for 1•4a

Crystal size: 602 × 162 × 92 μm^3, refined formula: $C_{132.77}H_{138.74}N_{24}O_{9.74}Zn_6I_{12}$, formula weight ($M_r$): = 4141.54, colorless block, crystal system: Monoclinic, space group *C*2, Z = 4, 32657 unique reflections merged from recorded 83543 ones (3.458° < θ < 76.137°) were used for structural analysis (R_{int} = 0.0606). Lattice parameters, *R*-factor on $F^2 > 2\sigma(F^2)$, weighted *R*-factor, goodness-of-fit, and Flack parameter (calculated from 11999 Parsons' quotients) are follows: a = 36.0217(6) Å, b = 14.6919(2) Å, c = 30.6728(7) Å, β = 101.672(2)°, V = 15897.2(5) Å^3, R = 0.0709, wR = 0.2050, S = 1.027, χ = 0.008(6). Calculated density is 1.730 g·cm^{-3}. Linear absorption coefficient (μ) is 19.759 cm^{-1}. Residual electron density (max/min) is 1.653/−1.161 eÅ^{-3}. CCDC number 1492161.

Structure analysis for 1•4a

The ORTEP diagram of the asymmetric unit of **1•4a** is shown in Figure S2. Four guest molecules were found in the asymmetric unit. One of the guest molecules (***A***) is ordered and has 100% occupancy. The other three guest molecules (***B***, ***C***, and ***D***) are disordered with solvent (cyclohexane) molecules. The occupancy of the guest at site ***B*** was estimated to be 72.9(16)% by least square refinement, and those of the guests at sites ***C*** and ***D*** were fixed at 50%, which were determined as their thermal parameters have reasonable values. Besides the guest and cyclohexane molecules, one molecule of diethyl phthalate (plasticizer) was found in the asymmetric unit, which was presumably derived from plastic apparatuses used in the experiment.

Some restraints should be applied for refinement of a disordered model. The geometries of the guests at ***B***, ***C***, and ***D*** are related with that of guest ***A*** with applying SAME (for the whole molecules), and all the guest molecules were refined with applying SIMU

(for the whole molecules).

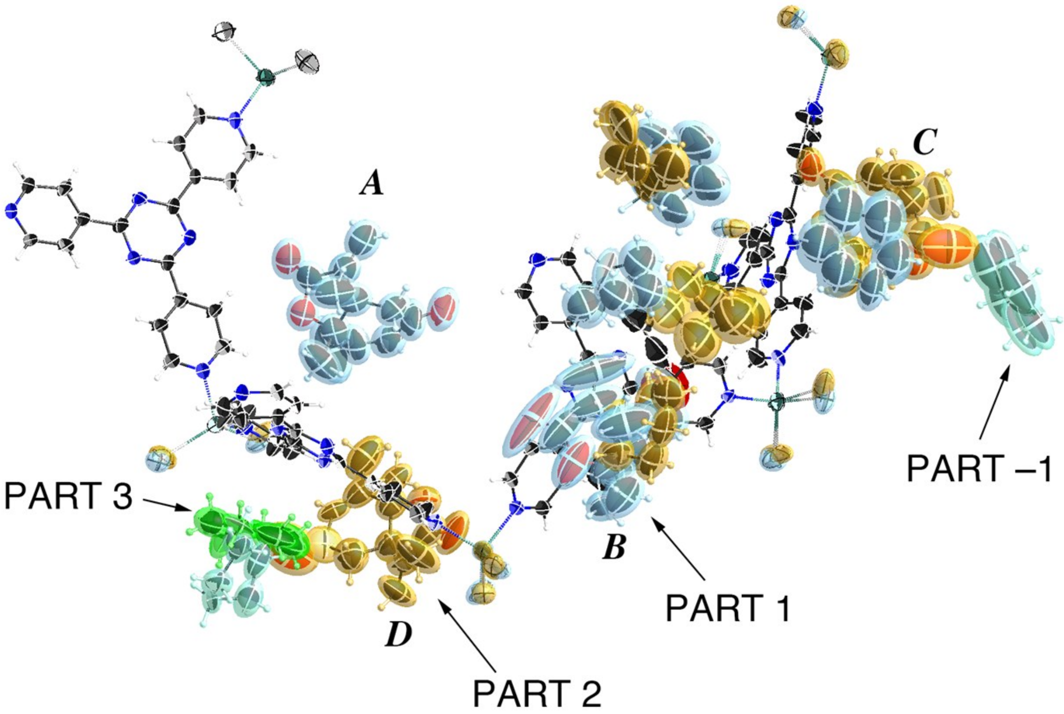

Figure S2. ORTEP diagram with 50% probability in the asymmetric unit of **1•4a**. Enclosed atoms by the PART command in SHELXL were represented using the difference color code.

3. Crystal 1•4b

Sample preparation for 1•4b

A solution of 5 μg of compound **4b** in 5 μL of 1,2-dichloroethan was added to a microvial including a crystal of crystalline sponge **1**. The microvial was sealed with a screw cap and incubated at 50 °C for 2 days. The resulting crystal was subjected to single crystal X-ray analysis.

Crystallographic data for 1•4b

Crystal size: 360 × 90 × 70 μm^3, refined formula: $C_{117.06}H_{104.25}N_{25.11}O_{10.05}Zn_6I_{12}$, formula weight ($M_r$): = 3938.54, colorless block, crystal system: Monoclinic, space group $C2$, $Z = 4$, 33613 unique reflections merged from recorded 72883 ones (1.963° < θ < 29.048°) were used for structural analysis ($R_{\text{int}} = 0.0297$). Lattice parameters, R-factor on $F^2 > 2\sigma(F^2)$, weighted R-factor, goodness-of-fit, and Flack parameter (calculated from 7518 Parsons' quotients) are follows: a = 34.9084(6) Å, b = 14.9228(2) Å, c = 29.6669(5) Å, β = 101.028(2)°, V = 15169.0(4) Å^3, R = 0.0604, wR = 0.1920, S = 1.012, χ = 0.059(20). Calculated density is 1.725 g·cm^{-3}. Linear absorption coefficient (μ) is 3.432 cm^{-1}. Residual electron density (max/min) is 1.341/−0.901 eÅ^{-3}. CCDC number 1492162.

Structure analysis for 1•4b

The ORTEP diagram of the asymmetric unit of **1•4b** is shown in Figure S3. Four guest molecules were found in the asymmetric unit. One of the guest molecules (***A***) is ordered and has 100% occupancy. Two guest molecules (***B*** and ***C***) are almost completely overlapped on each other, and their occupancies were fixed at 50%, which were determined as their thermal parameters have reasonable values. The other guest (***D***) is disordered with a solvent (cyclohexane) molecule, and its occupancy was estimated to be 61.0(10)% by least square refinement.

Some restraints should be applied for refinement of a disordered model. The geometries of the guests at ***B***, ***C***, and ***D*** are related with that of guest ***A*** with applying SAME (for the whole molecules), and all the guest molecules were refined with applying SIMU (for the whole molecules). In addition, DELU were applied in the refinement of the guest ***B*** (for the C_2=O and C_3–C_{3a} bonds), ***C*** (for the C_5=O, C_4–C_5, and C_5–C_6 bonds),

and ***D*** (for the C_5=O bond).

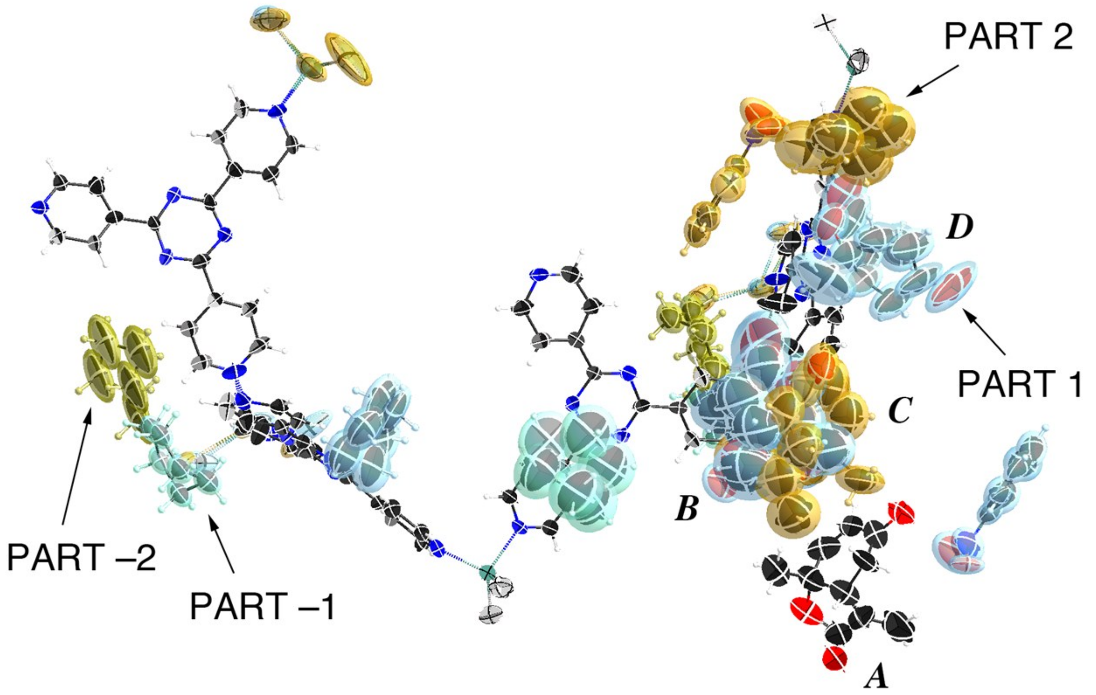

Figure S3. ORTEP diagram with 50% probability in the asymmetric unit of **1•4b**. Enclosed atoms by the PART command in SHELXL were represented using the difference color code.

4. Crystal 1•6

Sample preparation for 1•6

A solution of 5 μg of compound **6** in 5 μL of 1,2-dichloroethan was added to a microvial including a crystal of crystalline sponge **1**. The microvial was sealed with a screw cap and incubated at 50 °C for 2 days. The resulting crystal was subjected to single crystal X-ray analysis.

Crystallographic data for 1•6

Crystal size: 386 × 200 × 97 μm^3, refined formula: $C_{136.11}H_{131.89}N_{27.41}Zn_6I_{12}$, formula weight ($M_r$): = 4066.65, red block, crystal system: Monoclinic, space group $C2$, $Z = 4$, 35687 unique reflections merged from recorded 87712 ones (3.709° < θ < 76.010°) were used for structural analysis ($R_{\rm int}$ = 0.0558). Lattice parameters, R-factor on $F^2 > 2\sigma(F^2)$, weighted R-factor, goodness-of-fit, and Flack parameter (calculated from 10616 Parsons' quotients) are follows: a = 36.1897(8) Å, b = 14.6874(2) Å, c = 34.7427(8) Å, β = 109.742(2)°, V = 17381.5(6) Å^3, R = 0.0742, wR = 0.2219, S = 1.047, χ = 0.056(11). Calculated density is 1.554 g·cm^{-3}. Linear absorption coefficient (μ) is 18.024 cm^{-1}. Residual electron density (max/min) is 1.512/−0.997 eÅ^{-3}. CCDC number 1492163.

Structure analysis for 1•6

The ORTEP diagram of the asymmetric unit of **1•6** is shown in Figure S4. Seven guest molecules were found in the asymmetric unit. Three of the guest molecules (***A***, ***B***, and ***G***) are disordered with solvent (cyclohexane) molecules, and their occupancies were fixed at 75%, 33%, and 33%, respectively, which were determined as their thermal parameters have reasonable values. Two guest molecules (***C*** and ***D***) with their occupancies of 50% are overlapped on the symmetrically generated ones by the 2-fold rotation operation, and thus two guest molecules are disordered at each recognition site and occupy the inclusion sites ***C*** and ***D***, respectively, with the total occupancy of 100%. Two guest molecules (***E*** and ***F***) are largely overlapped on the guests at ***C*** and ***D***, respectively, and thus their occupancies are fixed at 50%.

Some restraints should be applied for refinement of a disordered model. The geometries of the guests ***B***–***G*** were related with that of the guest ***A*** with applying SAME (for the

whole molecules). In addition, all the guest molecules except ***E*** were refined with applying SIMU (for the whole molecules of the guests ***A***, ***B***, ***D***, ***F***, and ***G***, and for the benzene ring of ***C***).

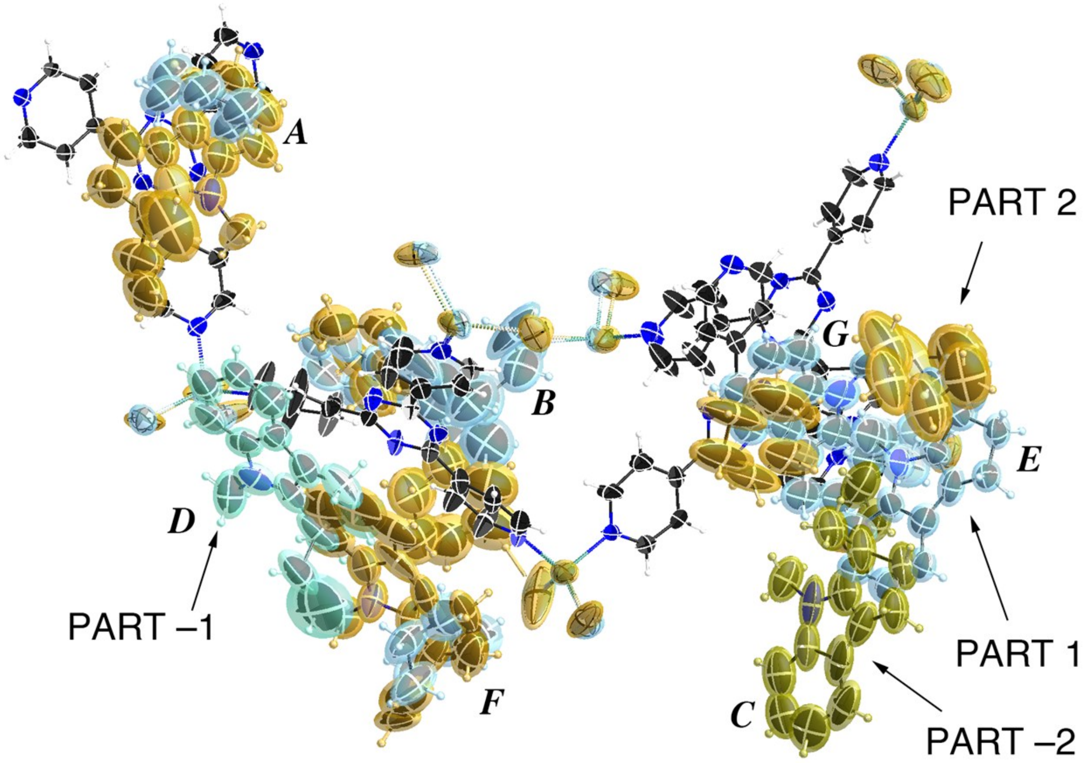

Figure S4. ORTEP diagram with 50% probability in the asymmetric unit of **1•6**. Enclosed atoms by the PART command in SHELXL were represented using the difference color code.

5. Crystal 1•8

Sample preparation for 1•8

A solution of 5 μg of compound **8** in 5 μL of 1,2-dichloroethane was added to a microvial including a crystal of crystalline sponge **1**. The microvial was sealed with a screw cap and incubated at 50 °C for 4 days. The resulting crystal was subjected to single crystal X-ray analysis.

Crystallographic data for 1•8

Crystal size: 262 × 163 × 85 μm^3, refined formula: $C_{100.85}H_{95.62}Cl_{11.35}N_{24.48}O_{2.88}S_{0.48}Zn_6I_{12.03}$, formula weight ($M_r$): = 4033.02, colorless block, crystal system: Monoclinic, space group $C2$, Z = 4, 28286 unique reflections merged from recorded 68884 ones (3.530° < θ < 68.247°) were used for structural analysis (R_{int} = 0.0696). Lattice parameters, R-factor on $F^2 > 2\sigma(F^2)$, weighted R-factor, goodness-of-fit, and Flack parameter (calculated from 6282 Parsons' quotients) are follows: a = 33.9973(10) Å, b = 14.9225(3) Å, c = 31.1158(8) Å, β = 101.047(3)°, V = 15493.3(7) Å^3, R = 0.0818, wR = 0.2560, S = 1.037, χ = 0.044(7). Calculated density is 1.729 g·cm^{-3}. Linear absorption coefficient (μ) is 22.084 cm^{-1}. Residual electron density (max/min) is 1.150/−1.371 eÅ^{-3}. CCDC number 1537112.

Structure analysis for 1•8

The ORTEP diagram of the asymmetric unit of **1•8** is shown in Figure S5. One guest molecule was found in the asymmetric unit. The guest molecule (***A***) is overlapped on the symmetrically generated one by the 2-fold rotation operation, and is disordered with four 1,2-dichloroethane molecules. The occupancy of the guest was estimated to be 47.9(8)% by least square refinement.

Some restraints should be applied for refinement of a disordered model. Two benzene rings are related with each other by applying SAME, and the guest molecule was refined with applying SIMU and RIGU (for the whole molecule). Two C=O bonds and two C–O–C angles of the ethyl ester groups are related with each other by applying SADI. The distances of four C–O bonds and seven C–C bonds and two Ph–CH_3 angles were fixed by applying DFIX and DANG. In addition, FLAT was applied to two toluyl groups. Considerably large void and residual unassignable electron densities with

amplitude of less than 1.0 $eÅ^{-3}$ on the d-Fourier map remained in the crystal structure, which indicates that the guest molecule including a heavy atom (sulfur) is no longer included in the void. We supposed that the void is filled with disordered solvent molecules, but we could not make a suitable model for the refinement of disordered solvent molecules due to messy electron density peaks. Therefore, the least square refinement at the last stage was performed using the reflection data modified by *PLATON*/SQUEEZE program.

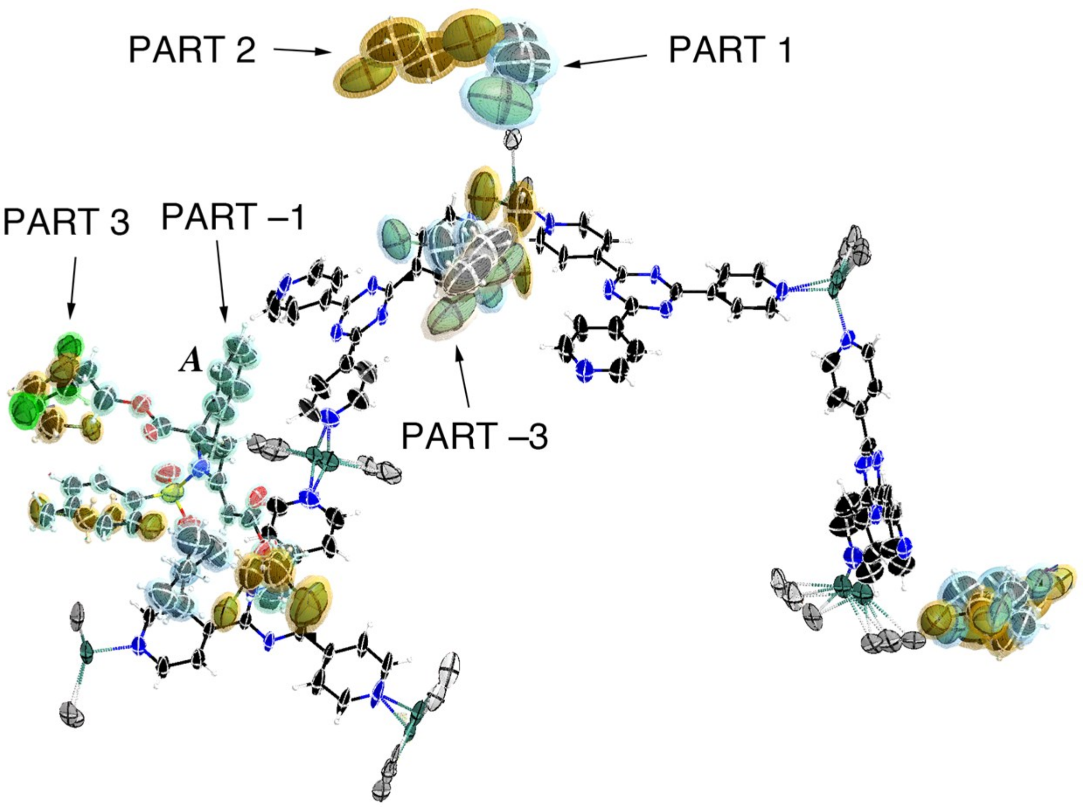

Figure S5. ORTEP diagram with 50% probability in the asymmetric unit of **1•8**. Enclosed atoms by the PART command in SHELXL were represented using the difference color code.

References

[1] G. M. Sheldrick, *Acta Crystallogr. Sect. A* **2015**, 71, 3-8.

[2] G. M. Sheldrick, *Acta Crystallogr. Sect. C* **2015**, 71, 3-8.
